# Effects of exercise on sex steroid hormones (estrogen, progesterone, testosterone) in eumenorrheic females: A systematic to review and meta-analysis

**DOI:** 10.1186/s12905-024-03203-y

**Published:** 2024-06-19

**Authors:** Wajiha Shahid, Rabiya Noor, Muhammad Salman Bashir

**Affiliations:** 1 Riphah College of Rehabilitation Sciences, Riphah International University, Lahore Campus, Lahore, 54600 Pakistan; 2 University of Management and Technology, Lahore, Pakistan

**Keywords:** Exercise performance, Follicular phase, Luteal phase, Menstrual cycle, Sex steroid hormones

## Abstract

**Background:**

The sex steroid hormones fluctuate during the menstrual cycle, which affects the strength and postural stability of females and leads to injuries and risk of falls. These hormones may be modulated by exercise to impact the overall health of females.

**Objective:**

To determine the effects of exercise on sex steroid hormones in eumenorrheic females.

**Methods:**

This review was performed following the Preferred Reporting Items for Systematic Reviews and Meta-Analyses(PRISMA) guidelines in Lahore, Pakistan. The full-length articles were searched using these databases/search engines (PubMed, Web of Science and Google Scholar, Sci-Hub). Randomized controlled trials along with single group experimental studies were also included. All types of exercises were compared with no exercise in the control group. The Cochrane Risk of Bias assessment tool assessed and screened the articles. The data were then analyzed. The primary outcomes were the levels of estrogen, progesterone and testosterone.

**Results:**

Eleven studies were included (5 randomized controlled trials and 6 quasi-experimental studies). The effects of exercise on free estradiol concentration and serum progesterone level were not significant [*p* = 0.37 (SMD = 0.33, 95% CI = 0.14 to 0.74, I^2^ = 0%) and *p* = 0.84 (S.D= -0.65, C.I= -6.92 to 5.62, I^2^ = 94%)] respectively, whereas, the effects on testosterone levels were significant [p value < 0.00001 (M.D = 0.89, 95% C.I= -2.16 to 3.95, I^2^ = 94%)].

**Conclusion:**

A blinded randomized controlled trial should be conducted in which a structured approach should be followed by women along with warm-ups, cool down and rest intervals.

**Trial registration number:**

The systematic review was registered prospectively on PROSPERO with registration number CRD42023473767.

**Supplementary Information:**

The online version contains supplementary material available at 10.1186/s12905-024-03203-y.

## Introduction

**Fig. 1:**
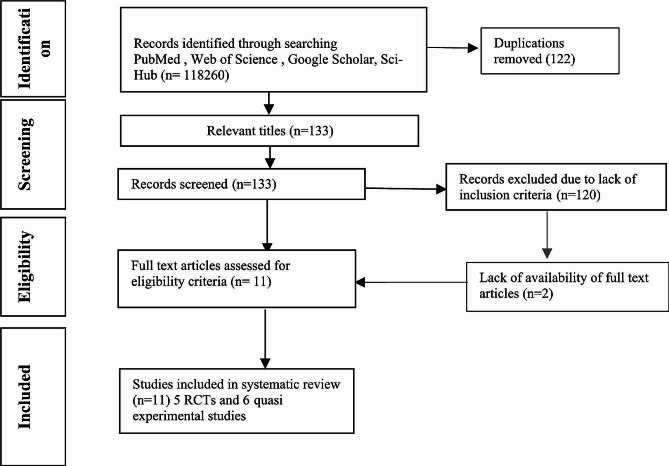
Flow chart of the study selection process

There are many specifications for differentiating females from males. Among these hormones are sex steroid hormones. These hormones include androgens (testosterone, androstenedione), estrogen (estradiol, estrone) and progesterone [1]. These hormones fluctuate periodically during the normal menstrual cycle (MC) and are presumed to have differential effects on muscle mass, strength and elasticity [2]. The MC consists of a series of phases that prepare the uterus for pregnancy. It lasts between 21 and 35 days and comprises follicular, ovulation and luteal phases. These main phases are divided into sub-phases, the early follicular, late follicular, ovulatory, early luteal, mid luteal and late luteal phases [3], as shown in Fig. 1.

The early follicular phase, 4 to 6 days, is characterized by low estrogen levels and progesterone with menstruation. This is followed by the late follicular phase in which estrogen increases, preparing the uterus for the ovulation phase. In the ovulation phase, there is a high estrogen and low progesterone concentration, leading to mature follicle rupture, which releases the egg [4]. The early luteal phase, following the ovulatory phase, is characterized by the corpus luteum’s formation and progesterone secretion. In the mid-luteal phase there is high estrogen and high progesterone, which prepares the endometrium for implantation of fertilized eggs. The luteal phase may end with pregnancy if the fertilized egg is implanted. If implantation fails the corpus will fail in the late luteal phase, and the endometrium will shed again, resulting in menstruation and menstrual cycle [3, 5].

Along with these hormonal changes basal body temperature can also be used to evaluate the phases of MC. A mid-cycle elevation of 0.2–0.3 degrees centigrade reflects the thermogenic action of increased progesterone levels [6]. Estrogen has a neuroexcitatory effect whereas progesterone has a neuroinhibitory effect [7]. This inhibitory and excitatory response is thought to cause alterations in power generation capacity, and joint laxity leads to variations in strength, power and joint sense during MC. Therefore, it is hypothesized that greater power generation occurs in the late follicular stage when progesterone is low, and estrogen is high. However, the luteal phase has a low power generation capacity when the progesterone level is high [3]. Testosterone elicits an excitatory response but there is no difference in bioavailable testosterone between the early follicular and mid-luteal phases [8]. The availability of this hormone in the ovulatory phase is unknown. It is well-established that physical performance or exercise affects sex steroid hormones and may be used in their modulation during MC. Menstrual cycle alterations are likely to occur after very demanding exercise, and this phenomenon has been observed and studied in females [9]. These exercises not only have physiological effects but are also known to have positive effects on the psychological behaviors of menstruating women, causing a decrease in menstrual pain and cramps [10].

Sex hormones play a vital role in female physiology and may be modulated by exercise. Despite the recognized importance of sex hormones in health and disease, the literature provides a fragmented understanding of how physical activity specifically impacts these hormones. Individual studies have explored the effects of various types, modalities, intensities, and durations of exercise on sex hormone levels in women. However, these studies exhibit considerable methodological diversity and inconsistent findings, posing challenges for drawing generalizable conclusions. Over a decade ago, a systematic review highlighted some preliminary associations but was limited by the scarcity and heterogeneity of studies. Since then, research in this area has expanded significantly, yet a comprehensive synthesis of these recent findings is conspicuously absent. Notably, no systematic review consolidating the burgeoning evidence has been published in the past ten years. Sex hormone levels serve as objective markers for evaluating the effectiveness and level of physical activity exposure. If exercise affects these levels, it could inform the type and dose of physical activity necessary for optimal health. Such insights are crucial not only for general health promotion but also for specific risk reduction strategies, particularly those targeting breast cancer, where hormonal mechanisms are pivotal. A preliminary literature search confirmed that no recent systematic reviews have thoroughly examined the impact of physical activity on sex hormone levels in healthy women, highlighting a critical gap. This underscores the necessity for an updated, methodologically rigorous synthesis of current evidence to advance understanding and guide future research and practice.

## Materials and methods

An evidence-based systematic research and meta-analysis followed the Preferred Reporting Items for Systematic Review and Meta-analysis (PRISMA) reporting guidelines. The 27-item checklist was followed [11]. Moreover, the protocol was prospectively registered in the PROSPERO database (International Prospective Register of Ongoing Systematic Reviews) with reference no CRD42023473767 [12].

### Eligibility criteria

The following inclusion criteria were used to recruit the articles: 1) the articles which were published in English.

### Population

The population of the study included: (a) The females without any menstrual dysfunction (eumenorrhea) (b) healthy females with no comorbidities e.g. cardiac issues, seizures etc… (c) females between the ages of 18 and 40 years.

### Intervention

Studies involving any type of exercise intervention such as strengthening exercises, aerobics or stretching exercises were included in the systematic review.

### Outcomes

Studies that included hormone levels (estradiol, progesterone and testosterone), strength, endurance and any 1 of these as an outcome measure were included.

### Study design

The studies included were randomized controlled trials and quasi-experimental studies available in English and full length. Moreover, articles from the last 10 years i.e. those published between 2012 and 2023 were included. This timeframe was selected to ensure that the review included recent studies that reflect current exercise interventions and methodologies and to capture advancements in research conducted over the past decade. Additionally, there was a systematic review of the studies before this time frame as there were a number of studies during this decade with better methodologies on which the systematic review was not performed so it was conducted.

### Search strategy

The full-length published articles were searched for systematic review in October 2023 using the following databases/ search engines: PubMed, Web of Science and Google Scholar and Sci-Hub. The PICO questions were then formulated as follows: P- population: eumenorrheic females, I- intervention: any kind of exercise (strengthening, endurance, mixed), C- comparator: any educational plan or no treatment at all, sedentary lifestyle, O- outcome: hormonal levels (estradiol, progesterone, testosterone), strength, postural stability, S- study design: experimental studies.

The first search was performed using the key terms ‘female AND exercises menstrual cycle’. There were 501 articles in PubMed, 135 in Web of Science, 17,500 in Google Scholar, and 101 in Sci-Hub. The next search was performed with the keyword ‘exercise eumenorrheic females’, which yielded 319 articles in PubMed and 6,140 articles in Google Scholar. The other keyword used was ‘exercise eumenorrheic female biomarkers’ which was used to search for 7358 articles in PubMed and 1310 articles in Google Scholar. A search was also performed using the keyword ‘exercise menstrual cycle strength’, which yielded 110 articles in PubMed, 56 in Web of Science, 66,100 articles in Google Scholar, and 145 in Sci-Hub. The next search was performed using ‘exercise eumenorrheic females’ strength’ as keyword which yielded 41 articles in PubMed and 3850 articles in Google Scholar. The keyword ‘exercise eumenorrheic females postural stability’ was used to search for 5 articles on PubMed and 1860 articles on Google Scholar. Finally, for ‘exercise menstrual cycle postural stability’ we obtained 212 articles from PubMed, 2 from Web of Science, 21500 from Google Scholar and 56 from Sci-Hub. This search was performed by the two reviewers independently. Several combinations of keywords were used to make the search effective (supplementary file).

All the included articles were subjected to screening under the following criteria.

### Data collection and analysis

### Selection of studies

The references identified through the search strategy were reviewed by one author (W.S.) in a two-step process. First, the title and abstract of each study were screened to exclude obviously ineligible studies. Second, the full texts of the remaining articles were examined and evaluated against predefined eligibility criteria. When necessary, a second review author (R.N.) was consulted. Additional information was requested from the study authors via email when needed.

### Data extraction

The study eligibility assessment, quality of the study, and risk of bias were performed following predefined inclusion criteria. Data extraction was performed by two co-authors. After removing the duplications, the full texts of the remaining articles were obtained and assessed on previously defined inclusion criteria for eligibility. In the event of disagreement, a third author evaluated and held a discussion with two authors to reach a consensus. The following information was extracted independently by both authors using the same form: author/publication year, study design, blinding, allocation concealment, number of patients(n), age, BMI, intervention arm, type of exercise given, comparator, study quality assessment outcome measure, tools, assessment period and number of sessions.

### Risk of bias assessment

**Fig. 2:**
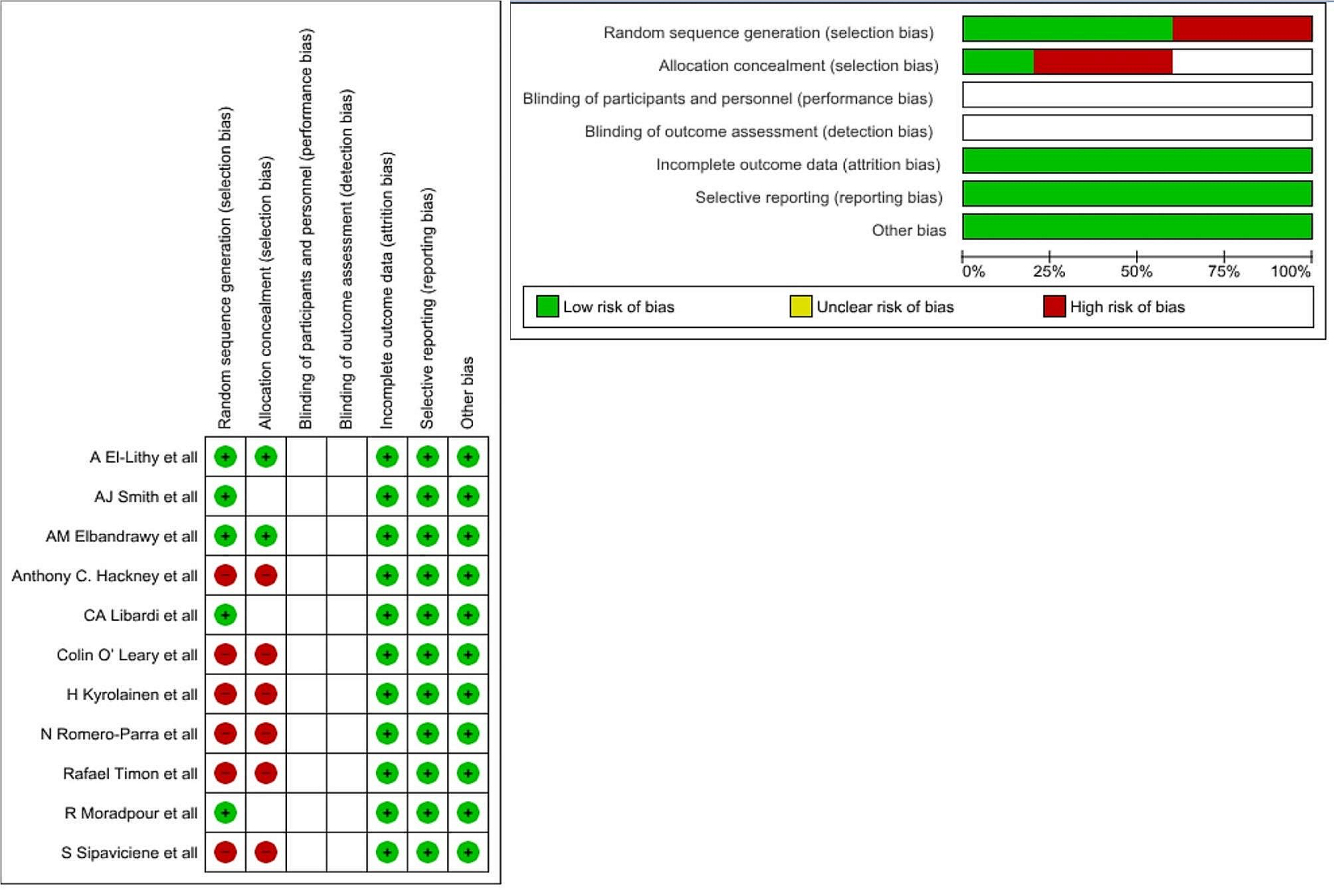
Risk of bias assessment graph and summary

The two reviewers individually assessed the risk of bias twice using the Cochrane risk of bias assessment tool, both for the risk of bias and for the overall risk of bias [13] as shown in Fig. 2. Sensitivity analyses were planned to explore the impact of different levels of risk of bias on the overall intervention effect. For quality assessment of the RCTs, the RoB_2.0 tool [14] was used on which 5 studies [15–19] were included. To determine the risk of bias of 6 quasi experimental studies 6 [20–25], all of which were single group studies, the ROBINS-1 tool [26] was used.

The following domains were used to assess the risk of bias: (1) Selection bias: random sequence generation and allocation concealment. (2) Performance bias: blinding of investigators, participants and care providers. (3) Detection bias: blinding of outcome assessment. (4) Attrition bias: incomplete data/differential dropout. (5) Reporting bias: selective reporting. (6) Other biases include conflicts of interest, follow-up, non-intention-to-treat, or per-protocol analysis. For each domain, the following descriptions were used to assess proper management of the risk of bias: ‘low risk,’ ‘high risk,’ or ‘unclear.’

### Measures of intervention effect and data synthesis

The quantitative studies’ data was synthesized using RevMan 5.4 software. The effect of exercise on estradiol, progesterone and testosterone was treated as a continuous variable. The mean difference, standard deviation and CI were derived using the RevMan software. When the effect of exercise was assessed multiple times in the study, the baseline and the end results were incorporated. For the quasii- experimental studies where there was no comparison group the pre-values were considered controlled values, whereas, the post-values were considered end result values.

The inverse variance method was used to combine the data from different studies and a random effect model was used to overcome the variability of the different studies. The mean difference was used for the studies in which the same unit of measure was used, whereas, the standard mean difference was used for the studies in which different units of measure were used. Heterogeneity was assessed by the I-squared test, which considered more than 50% relatively heterogeneous. The risk of bias in all studies was evaluated using the funnel plot method.

When two or more studies from the same cohort or data source were eligible for inclusion in the meta-analysis, we prioritized the following order: (1) the study with the highest quality score and the lowest risk of bias; [2] the study with the most informative measurements of effects on sex steroid hormones; [3] the study with a large sample size; [4] the study with the longest follow-up and [5] the study with the maximum adjustment for relevant potential confounding factors.

## Results

The systematic literature search yielded 118,260 records, 133 of which 133 had relevant titles. Of these 133 studies 122 were excluded because of their inclusion criteria. Eventually, 11 studies were included in the systematic review and meta-analysis: five were randomized controlled trials (RCTs) by Elbandrawy AM et al. (2021); Moradpour R (2019); El-Lithy A et al. (2015); Smith AJ et al. (2013); Libardi CA (2013) [15–19]and six were quasi-experimental studies Romero-Parra et al. (2020); Sipavičienė S et al. (2013); Timon R et al. (2013); Kyröläinen H et al. (2018), Hackney AC et al. (2020) and O’Leary C et al. (2013) [20–25] as shown in Fig. 1.

### Description of the studies

These 11 studies included randomized and nonrandomized trials comprising 584 females. Of the 584 females, 337 had a normal BMI, 17 were overweight and in 1 study, this was not mentioned. The mean age range across the studies was from 18.14 to 28.6 years and the mean BMI ranged from 18.7 to 25.7. Three studies of aerobic exercise [17–19], 3 studies of endurance [16, 22, 23], 2 studies of strengthening [22, 25], 1 study of isometric exercise [19], 1 study of isokinetic exercise [15], 1 study of eccentric exercise [20] and 1 study of stretch-shortening exercise loading [20] were used as intervention plan. There were 3 studies in which a sedentary lifestyle was used as a comparator [17–19], 1 in which vitamins B6 and supplements were used [16], 1in which isokinetic exercise was used [15] and 6 in which no comparator was used, as they were single group studies [20–25].

Seven studies reported estrogen levels as an outcome [16–18, 20–22, 25], five studies reported progesterone levels [16, 18–20, 25], and five studies reported testosterone levels [15, 22–25]. These outcomes were evaluated using blood and urine samples; 9 studies used blood samples [15, 16, 18–24], whereas 2 studies used urine samples [17, 25]. The duration of the session varied between single sessions and 4 months. The assessments were performed for testosterone levels immediately and at different times post-exercise in 3 studies [15, 23, 24]. In 5 studies the assessments were performed at pre- and post-exercise; in 4 studies progesterone was used as outcomes [16, 18, 19, 25], and in 4 studies, estrogen was used [16–18, 25], in 1 study pre-, mid- and post- level exercise estrogen and testosterone were assessed [22], in 1 study the baseline, 2 h, 24 h and 48 h post-exercise with levels of estrogen and progesterone were assessed [20] and in 1 study the baseline and various hours post-exercise estrogen levels were assessed [21]. Table 1 shows an overview of the characteristics and patient features of the selected studies. Two authors were contacted to obtain additional information, one of whom responded and the study was included.

**Table 1:** Overview of the characteristics and patient features of the selected studies

Author/ published in years	Elbandrawy AM et a l(2021)	Moradpour *R* (2019)	El-Lithy A et al. (2015)	Smith AJ et al. (2013)	Libardi CA (2013)	Romero-Parra et al. (2020)	Kyröläinen H et al. (2018)	Hackney AC et al. (2020)	Sipavičienė S et al. (2013)	Timon *R* et al. (2013)	O’Leary C et al. (2013)
Study design	RCT	RCT	RCT	RCT	RCT	quasi experimental study (single group)	quasi experimental study (single group)	quasi experimental study (single group)	quasi experimental study (single group)	quasi experimental study (single group)	quasi experimental study (single group)
No. of females	105	20	30	318	17	19	17	10	18	20	10
Mean age (year ± S.D)	22.40 ± 1.94	22.5 ± 1.9	18.14 ± 1.51	25.4 ± 0.3	22.5 ± 1.8	28.6 ± 5.9	27 ± 2	23.5 ± 2.1	20.2 ± 1.7	22 ± 0.1	20.0 ± 2.2
BMI	23.20 ± 1.71	18.7 ± 1.4	22.67 ± 2.34	24.8 ± 0.4	21.5 ± 1.4	22.3 ± 5.8	25.7 ± 4.6	21.8 ± 3.8	20.1 ± 2.5	21.7 ± 2.4	-------
Intervention	dynamic and active movements/ resistance exercises	running	treadmill run	treadmill, stair-stepper, or elliptical machine,	isokinetic exercise at slow velocity	squatting	spinning intervals with Body Bike indoor cycles and Techno Gym equipment/ free weight exercises	running	maximal drop jumps	intense training	treadmill run
Type of exercise	aerobic and isometric	aerobics	endurance	aerobics	isokinetic exercises	eccentric exercises	endurance and strength training	endurance	Stretch-shortening exercise loads	strength	endurance
comparator	sedentary lifestyle	sedentary lifestyle	vitamin B6 and Ca supplements	sedentary lifestyle	isokinetic exercise at fast velocity	nil	nil	nil	nil	nil	nil
Outcome measure	progesterone	estrogen, progesterone	estrogen, progesterone	estrogen	testosterone levels	estrogen, progesterone	estrogen, testosterone	testosterone levels	estrogen	estrogen, progesterone, testosterone	testosterone levels
Samples	blood	blood	blood	urine	blood	blood	blood	blood	blood	urine	blood
No of sessions	3 times/8 weeks = total of 24 sessions	3 times/8 weeks = total of 24 sessions	3 times/ week/3 months = total of 36 sessions	5 times/16 weeks = total of 80 sessions	5 set of 6 isokinetic exercises with 6 s rest interval between each set = single session	10 sets of 10 repetition at every phase of menstrual cycle = single session in each menstrual phase i.e. early follicular phase, late follicular phase and mid luteal phase,	3 times/ 9 weeks = total of 27 sessions	3 sessions in early follicular phase	100 rep/ 2 days of follicular and ovulation phase = single session in each phase	3 times/ 8 weeks = total of 24 sessions	60 min run at follicular and luteal phase of menstrual cycle = single session in each phase
Assessment period	pre and post assessment	pre and post intervention	pre and post treatment	pre and post treatment	baseline, immediately, 5, 15 and 30 min post exercise	baseline, 2 h, 24 h and48 h post exercise	pre, mid and post treatment	baseline, immediately, 30 min,60 min,24 h post-exercise	baseline and at various times up to 72 h post exercise	pre and post treatment	pre, immediately post and 30 min post exercise
Menstrual cycle phase	Follicular phase of menstrual cycle	Follicular phase of menstrual cycle	Luteal phase of menstrual cycle (day 20)	Mid-follicular phase (7–9 day of menstrual cycle) of menstrual cycle 2 and follow up menstrual cycle 6	---------	Early follicular phase, late follicular phase, mid luteal phase	---------	Early follicular phase of menstrual cycle	Follicular phase and ovulation phase of menstrual cycle	Menstrual phase, follicular phase, luteal phase	Mid follicular, mid luteal phase of menstrual cycle

No.=number, BMI = body mass index, nil = doesn’t exist

### Risk of bias in retained studies

According to the RoB_2.0 tool [14] for RCTs, 5 studies had low to moderate risk bias [16–19], and the remaining 1 had moderate to high-risk bias [15]. In all of these studies, the major factor contributing to the risk bias was “bias due to outcome measures” in which the blinding of the assessor was not mentioned and could have affected the results of the outcome parameters, as shown in Table 2.

**Table 2:** Quality assessment of RCT by RoB_2.0 tool [14]

Study	Selection bias	Performance bias	Detection bias	Attrition bias	Reporting bias	Overall
Smith AJ et al. (2013)	some concerns	Low	some concerns	high	Low	moderate
Libardi CA (2013)	low	some concerns	Low	high	Low	Some concerns
El-Lithy A et al. (2015)	Low	Low	Low	high	Low	Some concerns
Elbandrawy AM et a l(2021)	Low	Low	low	high	low	some concerns
Moradpour R (2019)	some concerns	low	Low	high	low	some concerns

According to the quasi-experimental studies, 5 had low-risk bias [21–25], while 1 had moderate risk bias [20]. Multiple factors contributing to bias were related to outcome factors, affecting the outcome domain, as shown in Table 3.

**Table 3:** Quality assessment for single group studies by ROBINS-1 tool [26]

Study	Confounding bias	Selection bias	Classification bias	Performance bias	Attrition bias	Detection bias	Reporting bias	Overall
Hackney AC et al. (2020)	low	low	Low	Low	low	low	low	low
O’Leary C et al. (2013)	low	low	low	Low	Low	Low	low	low
Kyröläinen H et al. (2018)	low	low	Low	low	Low	Low	Low	low
Timon R et al. (2013)	low	low	Low	Low	low	low	Low	low
Romero-Parra et al. (2020)	low	low	Low	Low	Moderate	low	Moderate	moderate
Sipavičienė S et al. (2013)	low	low	Low	low	low	low	low	low

The overall risk of bias varies across different studies and outcomes. While the RCTs shows some low to high risk bias particularly concerning the blinding and concealed allocation, non-randomized studies generally show a lower risk of bias as shown in Fig. 2. These assessments assume typical issues based on study types and should be refined with specific details from each study if discrepancies or particular issues were noted during the review process.

### Results of individual studies

Table 4 shows the age group of females ranging from 18 years to 28 years and the effect of exercise on sex steroid hormones. Sex steroid hormones are documented in different measuring units so due to the heterogeneity of the data it is difficult to interpret the effects of exercise on sex steroid hormones according to different age groups and menstrual cycles and to determine the dose-effect relationship.

**Table 4:** Overview of sex steroid hormone levels in response to exercise among different age groups and menstrual phases

Name of studies/year of publication	Mean age (year ± S.D)	Outcome measure	Before	After	Menstrual cycle phase
			Mean ± S.D	Mean ± S.D	
**Elbandrawy AM et a l(2021)**	22.40 ± 1.94	Progesterone in ng/ml	Aerobic = 7.47 ± 2.55 Isometric = 7.08 ± 3.40 Control = 7.61 ± 2.23	Aerobic = 10.95 ± 3.16 Isometric = 9.61 ± 2.80 Control = 7.72 ± 2.15	Follicular phase of menstrual cycle
**Moradpour R (2019)**	22.5 ± 1.9	Estrogen in ng/dl	Aerobics = 7.9 Control = 5.8	Aerobics = 9.8 Control = 6.5	Follicular phase of menstrual cycle
		Progesterone in ng/dl	Aerobics = 7.2 Control = 7.2	Aerobics = 7.2 Control = 7.3	
**El-Lithy A et al. (2015)**	18.14 ± 1.51	Estrogen in pg/dl	Study = 123.10 ± 39.85 Control = 107.30 ± 39.49	Study = 99.33 ± 36.33 Control = 108.70 ± 39.22	Luteal phase of menstrual cycle (day 20)
		Progesterone in ng/ml	Study = 9.73 ± 5.57 Control = 10.52 ± 5.22	Study = 5.65 ± 2.44 Control = 11.10 ± 5.03	
**Smith AJ et al. (2013)**	25.4 ± 0.3	Estrogen in nmol/d	Exercise = 7.8 (6.8–8.8) Control = 8.0 (7.0–9.1)	Exercise = 8.3 (7.2–9.5) Control = 8.0 (7.2–9.2)	Mid-follicular phase (7–9 day of menstrual cycle) of menstrual cycle 2 and follow up menstrual cycle 6
**Libardi CA (2013)**	22.5 ± 1.8	testosterone levels	------	-------	---------
**Romero-Parra et al. (2020)**	28.6 ± 5.9	Estrogen in pg/ml	------	38.2 ± 32.1, 185.1 ± 173.9 156.1 ± 91.5	Early follicular phase, late follicular phase, mid luteal phase
		Progesterone in ng/ml	-------	0.3 ± 0.1 0.4 ± 0.7 10.1 ± 3.9	
**Kyröläinen H et al. (2018)**	27 ± 2	Estrogen in pmol/L	453 ± 371	Post = 461 ± 455 After 4 weeks = 461 ± 271 After 9 weeks = 505 ± 355	---------
		Testosterone in nmol/L	2.25 ± 1.06	Post = 2.25 ± 1.06 After 4 weeks = 2.27 ± 1.11 After 9 weeks = 2.57 ± 1.87	
**Hackney AC et al. (2020)**	23.5 ± 2.1	testosterone levels in ng/dl	17.7^±^2.2	Post exhaustion = 27.6 ± 3.2 Post 30 min = 24.7 ± 2.0 Post 60 min = 22.4 ± 2.9 Post 90 min = 19.5 ± 2.2 Post 24 h = 13.9 ± 2.0	Early follicular phase of menstrual cycle
**Sipavičienė S et al. (2013)**	20.2 ± 1.7	Estrogen pmol/L	Ovulation phase = 229.6 ± 81.8 Follicular phase = 119 ± 61.7	------- --------	Follicular phase and ovulation phase of menstrual cycle
**Timon R et al. (2013)**	22 ± 0.1	Estrogen in ng steroid/mg	Menstrual phase = 15.3 ± 3.0 Follicular phase = 41.4 ± 8.9 Luteal phase = 23.2 ± 6.2	Menstrual phase = 12.4 ± 2.1 Follicular phase = 30.1 ± 7.2 Luteal phase = 23.4 ± 9.9	Menstrual phase, follicular phase, luteal phase
		Progesterone in ng steroid/mg	Menstrual phase = 13.8 ± 1.7 Follicular phase = 19.9 ± 3.0 Luteal phase = 38.3 ± 9.3	Menstrual phase = 10.6 ± 3.8 Follicular phase = 19.1 ± 5.2 Luteal phase = 28.0 ± 6.5	
		Testosterone in ng steroid/mg	Menstrual phase = 47.6 ± 18.4 Follicular phase = 48.5 ± 14.3 Luteal phase = 52.5 ± 10.7	Menstrual phase = 47.4 ± 11.1 Follicular phase = 51.1 ± 15.0 Luteal phase = 50.5 ± 16.3	
**O’Leary C et al. (2013)**	20.0 ± 2.2	testosterone levels in nmol/L	Mid-follicular = 1.41 ± 0.21	Mid-follicular Post 1.86 ± 0.21 30 min rest1.75 ± 0.32	Mid follicular, mid luteal phase of menstrual cycle
			Mid-luteal = 1.27 ± 0.23	Mid luteal Post 2.43 ± 0.56 30 min rest = 1.69 ± 0.34	

### Synthesis results

#### Effect of exercise on estradiol

**Fig. 3:**
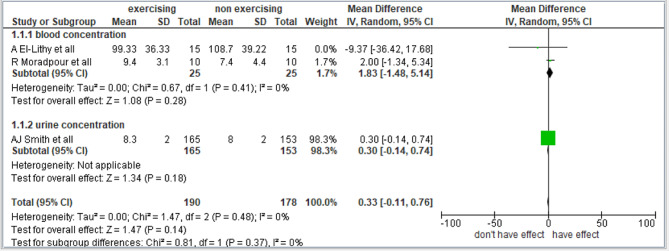
Forest plot of the comparison of “no exercise” vs. “any kind of exercise” outcomes: free estradiol concentration in blood and free estradiol concentration in urine. C.I: confidence interval, M.D: mean difference

Three studies were included to determine the effect of exercise on estradiol [16–18]. The subgroup analysis was performed in 2 studies by blood concentration; the remaining study used urine concentration. There were 368 participants (190 in the experimental group and 178 in the control group). Quasii- experimental studies [20, 21, 25] were excluded from the analysis. The overall effect of exercise on the concentration of estradiol was found not significant (*n* = 3, SMD = 0.33, 95% CI = 0.14 to 0.74, I^2^ = 0%). The forest plot was almost asymmetrical, as shown in the Fig. 3.

### Measure of heterogeneity

I^2 = 0%: This shows that all the studies included were homogeneous with all the observed variability due to sampling variance. This sampling variability may be due to differences in the mean age(in years) of the females 18.14 ± 1.51years [16], 22.5 ± 1.9 years [18] and 24.8 ± 0.4 years [17].

Tau² (τ^2 = 0.00): This value represents the estimated amount of total heterogeneity. The square root of τ^2 (τ = 0.00) indicates the average deviation of study effects from the pooled effect size, highlighting substantial variation across the sample of the studies.

### Sensitivity analysis

EL-Lithy A et al. (2015): a randomized controlled trial (RCT) involving endurance exercises, reported a decrease in estrogen levels from 123.10 to 99.33 pg/dl.

Moradpur R (2015): a randomized controlled trial (RCT) involving aerobics exercises, reported an increase in estrogen levels from 7.8 to 9.8 ng/dl.

Smith AJ et al. (2013): a randomized controlled trial (RCT) involving aerobics exercises, reported an increase in estrogen levels from 7.8 to 8.3 nmol/d.

The studies had substantial heterogeneity due to the use of different measuring units.

### Subgroup analysis results

### Endurance exercises

- Mean difference in testosterone levels: 23.77 pg/dl.
- Pooled Standard Deviation: Not applicable (only one study in this subgroup).
- Number of Studies: 1.

### Aerobics exercises

- Mean difference in testosterone levels: Not applicable (due to heterogeneity in the data).
- Pooled Standard Deviation: Not applicable (due to heterogeneity in the data).
- Number of Studies: 2.

### Funnel plot

**Fig. 4:**
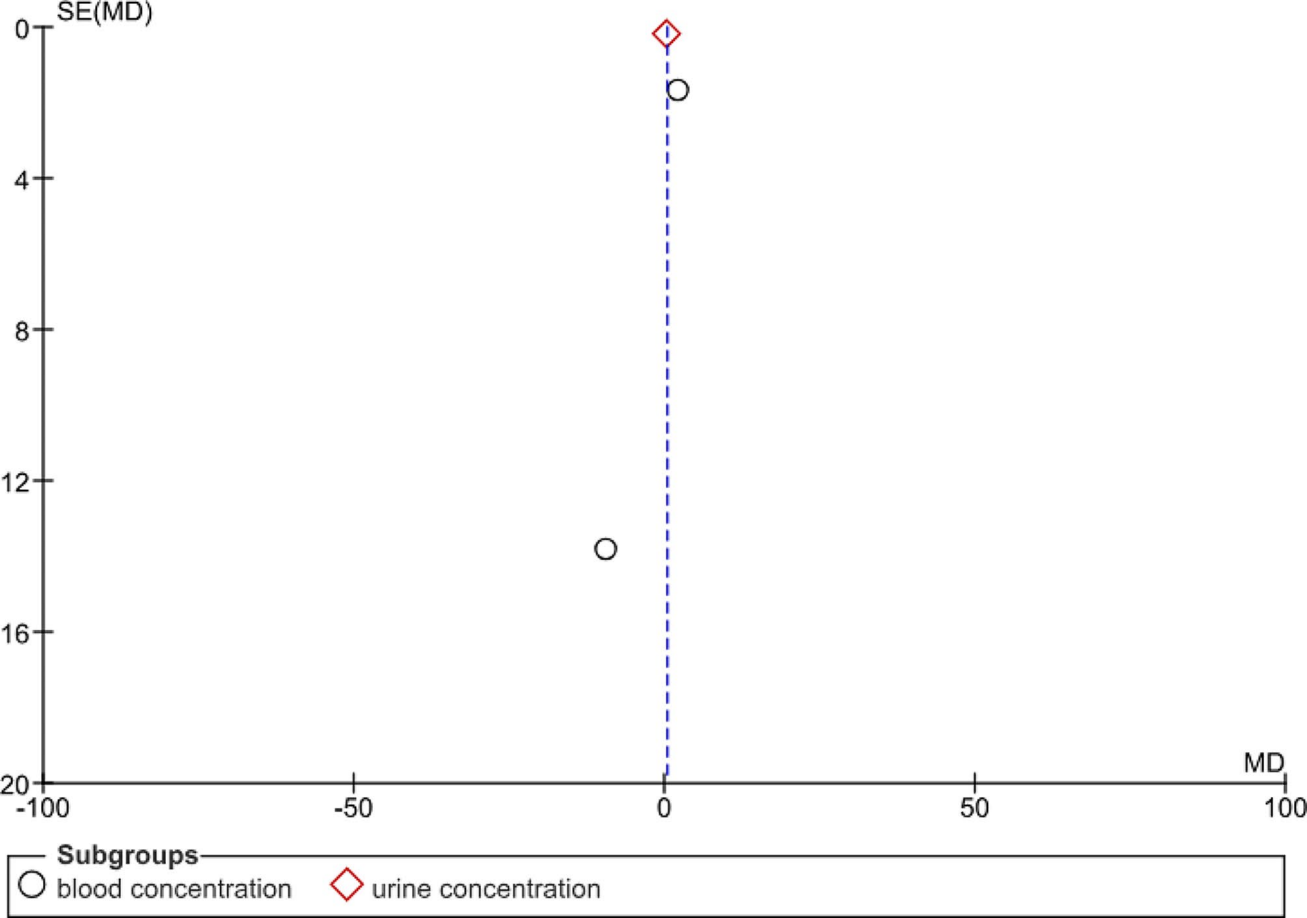
Funnel plot of standardized mean differences in estrogen levels versus the precision of the studies

The funnel plot created for the standardized mean differences in estrogen levels versus the precision of the studies (inverse of standard error) is displayed in Fig. 4. Ideally, the plot should resemble an inverted symmetrical funnel without publication bias, with studies scattered evenly around the mean effect line. Given our small number of studies and the lack of visible asymmetry, there was no immediate indication of severe publication bias from the plot alone. However, due to the small sample size, this interpretation should be cautiously approached.

### Results of Egger’s test

The results of Egger’s test, which are more quantitative, suggest that the regression coefficient for the standard error is not statistically significant (note: with such a small sample size, these results are not very reliable). This test aims to detect funnel plot asymmetry by regressing the standard normal deviation (effect size divided by its standard error) against the precision (inverse of the standard error). A nonsignificant coefficient implies no strong evidence of publication bias, but again, the number of studies limits the reliability.

#### Effect of exercise on progesterone

**Fig. 5:**
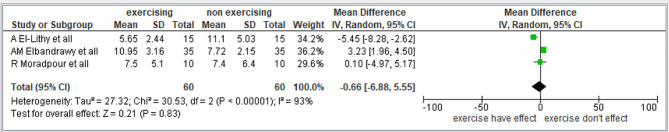
Forest plot of comparison “no exercise” vs. “any kind of exercise” outcome: serum progesterone concentration in blood. C.I: confidence interval, M.D: mean difference

The review included three studies to evaluate the effect of serum progesterone levels after physical activity [16, 18, 19]. Quasi-experimental studies were excluded [20]. The overall effect was nonsignificant (*n* = 3, SD.= -0.65, CI.= -6.92 to 5.62, I^2^ = 94%). The forest plot was roughly symmetrical, as shown in the Fig. 5.

### Measure of heterogeneity

I^2 = 93%: This extremely high value suggested substantial heterogeneity among the studies included in the meta-analysis. This indicated that 94% of the variability in effect estimates is due to differences between studies rather than sampling errors within studies.

Tau² (τ^2 = 27.32): This value represents the estimated amount of total heterogeneity. The square root of τ^2 (τ = 5.22) represented the average deviation of the study effects from the pooled effect size, highlighting substantial variation across the studies.

### Sensitivity analysis

EL-Lithy A et al. (2015): a randomized controlled trial (RCT) involving endurance exercises reported a decrease in progesterone levels from 9.73 to 5.65 ng/ml.

Elbandrawy AM et al. (2021): a randomized controlled trial (RCT), reported an increase in progesterone levels from 7.47 to 10.95 ng/ml for aerobic exercise and from 7.08 to 9.61 ng/ml for isometric exercise.

Moradpur R (2015): a randomized controlled trial (RCT) involving aerobics exercises, reported no change in progesterone levels.

There was heterogeneity in the data, with 1 study showing an increase in progesterone levels, the other showing a decrease and the third showing no change.

### Subgroup analysis results

### Endurance exercises

- Mean difference in progesterone Levels: 4.08 ng/ml.
- Pooled Standard Deviation: Not applicable (only one study in this subgroup).
- Number of Studies: 1.

### Isometric exercises

- Mean difference in progesterone Levels: 2.53 ng/ml.
- Pooled Standard Deviation: Not applicable (due to one study in the data).
- Number of Studies: 1.

### Aerobics exercises

- Mean difference in progesterone Levels: 3.48 ng/ml.
- Pooled Standard Deviation: 0.77.
- Number of Studies: 2.

There was variability in the mean differences among the studies.

### Funnel plot

**Fig. 6:**
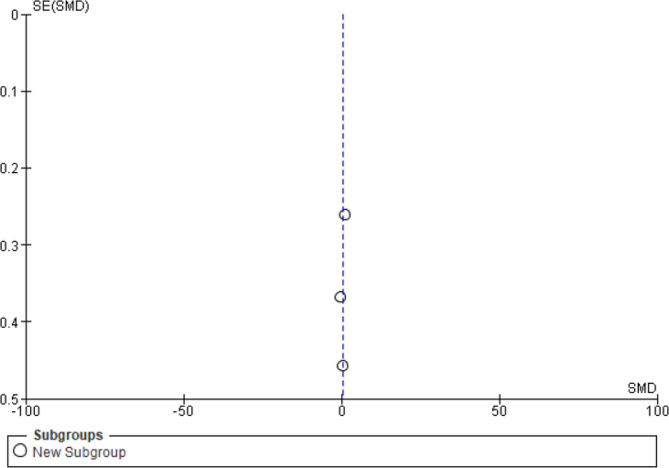
Funnel plot of standardized mean differences in progesterone levels versus the precision of the studies

A funnel plot was created for the standardized mean differences in progesterone levels versus the precision of the studies (inverse of standard error) as shown in Fig. 6. Due to our small number of studies and the lack of visible asymmetry, there was no immediate indication of severe publication bias from the plot alone.

### Results of Egger’s test

The results of Egger’s test suggest that the regression coefficient for the standard error is not statistically significant (note: with such a small sample size, these results are not very reliable).

#### Effect of exercise on testosterone

**Fig. 7:**
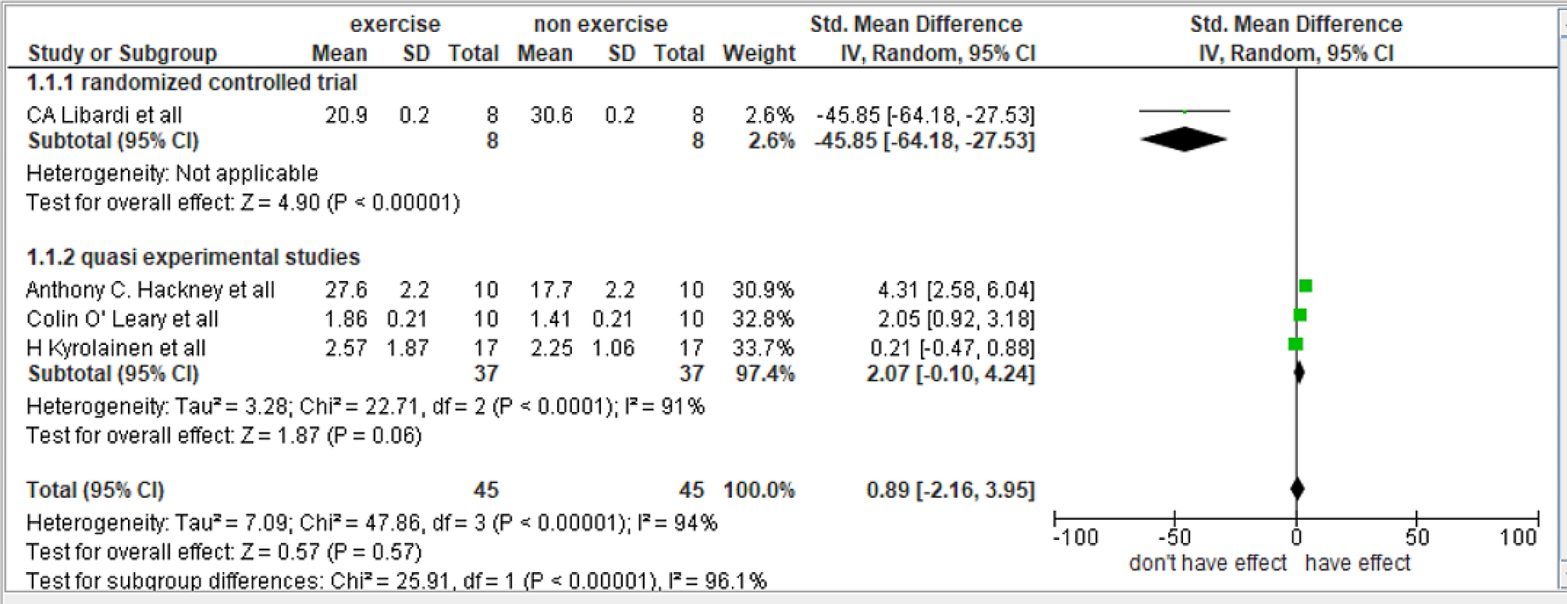
Forest plot of the comparisons of “no exercise” vs. “any kind of exercise” outcomes: testosterone concentration in blood. C.I: confidence interval, SDM: standard mean difference

Four studies were included in the meta-analysis to determine the effect of exercise on circulating testosterone [15, 22–24]. Due to the scarcity of data, a subgroup was performed to include the quasi- experimental studies. The overall effect was significant (*n* = 4, MD.=0.89, 95% CI.= -2.16 to 3.95, I^2^ = 94%). The forest plot was roughly symmetrical as shown in Fig. 7.

### Measure of heterogeneity

I^2 = 94%: This extremely high value suggested substantial heterogeneity among the studies included in the meta-analysis. This indicated that 94% of the variability in effect estimates is due to differences between studies rather than sampling errors within studies.

Tau² (τ^2 = 7.09): This value represents the estimated amount of total heterogeneity. The square root of τ^2 (τ = 2.66) described the average deviation of the study effects from the pooled effect size, highlighting substantial variation across the studies.

### Sensitivity analysis

- **Libardi CA (2013)**, a randomized controlled trial (RCT) involving isokinetic exercises, reported increased testosterone levels from 2.5 to 3.0 nmol/L.
- **Hackney AC et al. (2020)**, a quasi-experimental study with endurance exercises, demonstrated an increase from 1.8 to 2.3 nmol/L.
- **O’Leary C et al. (2013)**, another quasi-experimental study with endurance exercises, showed an increase from 2.0 to 2.5 nmol/L.
- **Kyrolainen H et al. (2018)**, a quasi-experimental study focusing on strength exercises, showed an increase from 2.2 to 2.7 nmol/L.

For each study, the mean difference in testosterone levels due to the exercise intervention was 0.5 nmol/L.

### Subgroup analysis results

#### Endurance exercises

- Mean difference in testosterone Levels: 0.5 nmol/L.
- Pooled Standard Deviation: Virtually zero (indicative of consistent results across studies).
- Number of Studies: 2.

#### Isokinetic exercises

- Mean difference in testosterone Levels: 0.5 nmol/L.
- Pooled Standard Deviation: Not applicable (only one study in this subgroup).
- Number of Studies: 1.

#### Strength exercises

- Mean difference in testosterone Levels: 0.5 nmol/L.
- Pooled Standard Deviation: Not applicable (only one study in this subgroup).
- Number of Studies: 1.

The results showed that regardless of the type of exercise (endurance, isokinetic, strength), the mean difference in testosterone levels postexercise intervention was consistent at 0.5 nmol/L across all types of exercise. This suggests a uniform response in testosterone levels to different types of physical activities in the studies considered.

### Funnel plot

**Fig. 8:**
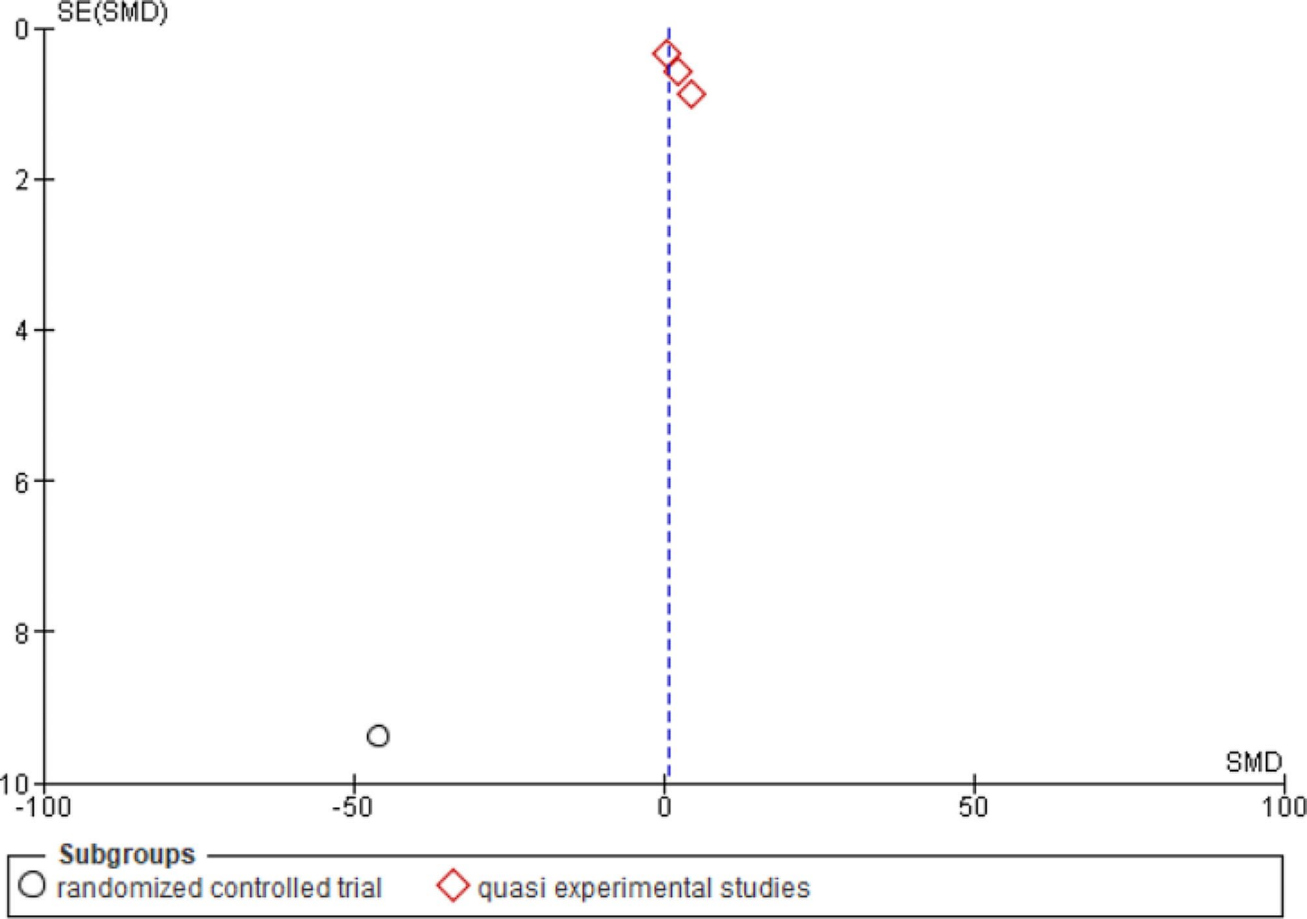
Funnel plot of standardized mean differences in testosterone levels versus the precision of the studies

The funnel plot created for the standardized mean differences in testosterone levels versus the precision of the studies (inverse of standard error) is displayed in Fig. 8, showing no serious concerns about publication bias.

### Results of Egger’s test

The results of Egger’s test, which are more quantitative, suggest that the regression coefficient for the standard error is not statistically significant (note: with such a small sample size, these results are not very reliable).

## Discussion

Sex steroid hormones play an important role in female physiology, mainly by regulating muscle mass and function [27], contributing to sex-related differences in cardiovascular function [28], influencing sexual differentiation of the central nervous system, influencing female reproductive cancers [29] and regulating estrogen receptors in airway smooth muscle cells [30]. These hormones also play an important role in the physical performance of athletes [31], for which this topic has gained importance, and multiple exercises have been planned to determine the effects of these hormones.

Therefore, after passing through the filtering criteria of the review, 11 studies were included in this systematic review and meta-analysis, of which five were randomized and six were nonrandomized controlled trials. Articles from the last 10 years were searched, but there was a scarcity of data, as few randomized controlled trials were found. Due to their scarcity, nonrandomized controlled rials were also included in the review. Sex steroid hormones are influenced by exercise, irrespective of the mode of exercise. However, these effects were not found to be significant, and the data were heterogeneous. Moreover, most of the studies considered these effects only in the late luteal phase.

Free estradiol is considered to increase after exercise, with little effect on total estradiol levels [18]. These estradiol levels are considered to positively affect the strength and endurance of females [32], which increase in the ovulation phase and decrease in the late luteal phase, leading to postural sways and a risk of injuries in athletes [33]. However, due to exercise, the estradiol level in the blood is increased, leading to an overall increase in well-being, strength and risk of injury. These levels are considered to decrease in the urine, possibly due to the retention of hormones in the blood. This finding is consistent with prior studies [17]. However, in some studies, it has also been reported that exercise leads to lower estradiol levels [16]; therefore, due to this heterogeneity of data, which may be due to overall characteristics (age, BMI, mode of exercise), a consensus cannot be reached on the overall effects, and more studies should be conducted.

Serum progesterone levels are said to increase after exercise [19]. Progesterone levels are considered to increase after the middle cycle in the early ovulation phase, during which the body is prepared for conception [19]. By increasing the levels of progesterone through exercise, the chances of pregnancy may increase. However, in some studies, the level of progesterone is said to decrease, leading to improvement in the symptoms of fatigue and heterogeneity of the data [16].

Testosterone levels increase immediately after exercise and decrease over time [23]. These levels of testosterone are considered to have a positive effect on the strength of females, which is consistent with previous studies related to males [34]. This hormone is found in trivial amounts naturally in females, but exercise increases testosterone levels, which are considered to have a positive effect on the strength and anti-inflammatory responses of females temporarily [35].

As far as the secondary outcomes are considered, the weight of the females decreased, and heath improved, with marked improvement in premenstrual syndrome symptoms, i.e., pain, menstrual cramps and agitation [36]. Moreover, there were significant effects on body mass index, total fat mass and waist circumference. There are also effects on other hormones, such as cortisol and growth hormones [22], as well as increases in hemoglobin, hematocrit, red blood cell count and platelet count, increasing the overall health status of females [16].

High-intensity exercise is considered to disturb the menstrual cycle [37], while moderate aerobic exercise tends to impact the overall well-being of females positively. However, the exercise mode, intensity and dosage tended to create heterogeneity in the studies. Although few randomized controlled trials have been conducted in the last 10 years, all of them lack any proper warm-up or cool-down regimens or rest intervals; moreover, the majority of them have a high risk of bias of outcome, as no blinding was documented for the assessor. This may have led to bias in the outcome results. Therefore, there is a need to conduct a randomized controlled trial with proper blinding to decrease the risk of bias along with the proper regimen to follow, which may be followed unanimously to have a positive effect on the hormonal level of females, ultimately reducing the effects of premenstrual syndrome, increasing the risk of conception and decreasing the effects of other systemic issues, such as cardiac [38] and osteoarthritis [39], and premenopausal syndrome leading to primary prevention [40].

## Conclusion

Over the last 10 years, several randomized controlled trials have been conducted to determine the effects of exercise on the hormonal profiles of eumenorrheic females. These studies have shown that exercise has an impact on the sex steroid hormones of females. Still, due to the heterogeneity of the data, it is difficult to determine the overall effect on estradiol, progesterone and testosterone. Moreover, most of the studies have considered the effects in the late luteal phase, with no comparison between the different phases of the menstrual cycle. Therefore, a good randomized controlled trial should be conducted with proper treatment regimens to determine the appropriate treatment effects.

### Study limitations

The current study has several limitations. The studies included in this meta-analysis had significant heterogeneity that affected the validity of the results. Second, there were limited studies available, including randomized control trials and nonrandomized trials, and some of the studies had small participant numbers, which limits the generality of the results. Third, due to the small number of available studies, both high- and low-Pedro score studies were included, which affected the treatment effect size. Finally, only studies that were published in the English language were included, resulting in bias.

Future RCTs should include larger sample sizes and optimal standardized therapy protocols for both the exercise and control groups. Ample strategies should be taken to limit confounding factors such as dietary intake and psychological status. Moreover, a standard protocol should be formulated for a diverse range of females, and a unified exercise plan should be provided to document the effects of these interventions on sex steroid hormones.

### Recommendations for further research

The number of studies included in the meta-analysis should be increased to provide a more robust assessment of publication bias. Additional studies would help stabilize the funnel plot and provide more reliable results from Egger’s test. Moreover, given the significant heterogeneity and the confirmed positive effect of exercise on sex steroid hormones, it is crucial to explore the sources of this heterogeneity in future research. Potential sources could include differences in exercise modalities (aerobic vs. resistance), participant demographics (age, baseline fitness level), and timing of hormone measurements relative to exercise sessions.

### Electronic supplementary material

Below is the link to the electronic supplementary material.

Supplementary Material 1

## Data Availability

The datasets used and/or analyzed during the current study will be available from the corresponding author upon reasonable request, according to the research policies of the university from which ethical approval has been obtained for this study.
