# Supplementary material for: Effects of exercise on sex steroid hormones (estrogen, progesterone, testosterone) in eumenorrheic females: A systematic to review and meta-analysis
Source: BMC Womens Health. 2024 Jun 19;24:354. doi: 10.1186/s12905-024-03203-y (PMC11186217; doi:10.1186/s12905-024-03203-y)
Supplement: Supplementary file 1 — Supplementary Material 1 [file 12905_2024_3203_MOESM1_ESM.docx]

Combinations of keywords used for search

| Search strategy for MEDLINE |
| --- |
| Number Search items     1. female / exercises menstrual cycle 2. exercise / eumenorrheic females 3. exercise / eumenhorreic / females biomarkers 4. exercise / menstrual cycle / strength 5. exercise / eumenhorreic females / strength 6. exercise / eumenhorreic females /postural stability 7. exercise / menstrual cycle / postural stability 8. physical activity / sex steroid hormones 9. physical activity / estrogen/ progesterone 10. physical activity / testosterone 11. physical training / sex steroid hormones 12. physical training/ estrogen/ progesterone 13. physical training / testosterone 14. 1 to 3 15. 4 to 7 16. 3 and 5 |
